# The periodical formation of the vaginal closure membrane in guinea pigs is a regeneration process maintained by a special stem cell niche

**DOI:** 10.21203/rs.3.rs-10195384/v1

**Published:** 2026-07-20

**Authors:** Shanshan Wang, Zhengui Zheng

**Affiliations:** Southern Illinois University School of Medicine; Southern Illinois University School of Medicine

**Keywords:** vaginal closure membrane, periodical formation, regeneration process, adult guinea pigs, adult stem cells

## Abstract

In adult females of most hystricomorph rodents (animals in the suborder Hystricomorpha, such as guinea pigs), the vagina is open only during heat period; for the remainder of the estrous cycle, it is occluded by a structure resembling the human hymen—known as the “vaginal closure membrane” (VCM). This process of periodic formation and regression of the VCM persists throughout the animal’s entire life, from puberty until death. To date, the underlying mechanisms explaining why only this small subset of rodents periodically forms a VCM remain largely unelucidated. In this study, we compared the epithelium structure around the external vaginal orifice between guinea pigs and mice, revealed that a hairless epidermal transition zone exists in guinea pig but not mice. The proliferation of keratin (Krt) 10-positive epithelial cells in this zone plays key roles in VCM formation. Hormone treatment in ovariectomized guinea pigs revealed progesterone may promote the formation and thickening of VCM. Ki67 cytoplasmic localization and strong expression of progenitor cell marker Krt19 in epithelial cells at external vaginal orifice of guinea pigs but not mice indicated that the VCM formation in guinea pigs is most likely a tissue remodeling process. And in addition, we identified a novel population of stem cells expressing pluripotency markers at the external vaginal orifice of guinea pigs. Our findings suggest that, in guinea pigs, pluripotent stem cells may reside within the epithelial tissue of the vaginal opening region of adult females and is responsible for VCM formation.

## Introduction

It has been reported that humans and various other mammals possess a hymen^1^. In most females of the suborder Hystricomorpha (commonly known as hystricomorph rodents), including guinea pigs, an occluding membrane presents at the vaginal orifice not only during the juvenile and immature stages, but throughout the female’s lifetime; this membrane also periodically ruptures prior to the onset of estrus in each estrous cycle, or following parturition, only to subsequently reform^2–4^. Unlike the hymen found in humans, the membrane at the vaginal opening of a female guinea pig remains completely closed most of the time; however, it opens spontaneously during each estrus in estrous cycle, requiring no male involvement^3,5^. As early as 1892, Retterer reported that the vaginal mucosa of non-pregnant guinea pigs undergoes cyclical changes—specifically, the formation of an epithelial membrane that occludes the vaginal orifice^6^. This completely closed membrane was first named the “vaginal closure membrane” (VCM) by Stockard and Papanicolaou in 1917; subsequently, the authors reported that the VCM persists throughout the entire gestational period and opens at the time of delivery^5,7^. Further study suggested that the periodical opening and closing of the vaginal orifice was correlated with the time of estrus^8^.

Following each estrus and/or parturition, the formation of VCM is completed within a short period. Observations indicate that VCM becomes clearly visible within 2 to 3 days after mating or parturition^2,3^. In sexually mature female guinea pigs, the VCM remains open for 1 to 3 days during estrus; conversely, during proestrus, metestrus, and diestrus, the vaginal orifice remains closed, and a pale VCM can be easily observed^9^. Furthermore, the VCM in adult females was reported to be regulated by ovarian hormones, however, even after ovariectomy, the membrane continues to form, subsiding only upon the administration of estrogen^10,11^. Consequently, it remains unclear precisely which specific hormone is involved in the formation of the VCM. Literature regarding VCM is extremely limited, with the majority focusing on the opening of the vaginal orifice; only a very small number of histological studies have addressed the formation process of VCM^2,5,12^. The early observations found that the formation of the VCM occurs due to post-inflammatory healing process^12^. A recent study focusing on Galea spixii-another species within the Caviidae family, suggested that the formation of this membranous structure might be attributed to cell proliferation within the germinative stratum layer, as well as the shedding of the superficial keratinized layer resulting from the mutual adhesion of cells on the opposing walls of the vaginal canal^2^. To date, we know nothing about the mechanism by which the adult females of hystricomorph rodents periodically form VCMs, a phenomenon not observed in other mammals.

Comparative analysis plays a crucial role in elucidating the mechanisms of regeneration and wound healing by highlighting the differences—at the molecular, cellular, and genetic levels—between tissues that heal via regeneration (scarless) and those that heal via repair (scar-forming)^13^. By comparing different species, tissue types, or treatment methods, researchers are able to identify the key pathways that determine the ultimate quality of healing tissue—such as immune responses, angiogenesis, and extracellular matrix remodeling^14^. Much of the foundational knowledge in regenerative medicine is derived from comparisons among various mammals, or from comparing them with animals possessing strong regenerative capabilities, such as salamanders^15–17^.

Stem cells are capable of modulating inflammatory responses, accelerating wound closure, promoting angiogenesis, and guiding tissue remodeling; by differentiating into specialized cells and releasing therapeutic growth factors, they serve as the body’s internal repair system^18–21^.

Adult stem cells have currently been identified in various tissues, including blood, the gut, skin, muscle, the brain, and the heart; furthermore, numerous studies have confirmed that these adult stem cells, derived from different organs, possess the capacity to repair and regenerate their respective organs^22–24^. Vaginal epithelium is a stratified squamous epithelium consisting of three layers: basal layer, suprabasal layer, and apical cornified layer^25^. During embryonic development, the vaginal epithelium exhibits a dual origin: its lower portion is derived from the urogenital sinus (endoderm), while its upper portion originates from the Müllerian ducts (mesoderm); this characteristic is observed in various species, including humans, mice, and guinea pigs^26–28^. After a complicated developmental process involving cell proliferation, transformation, and interaction with underlying tissues, leading to the adult stratified squamous lining^29^. Most epithelia need to constantly replace damaged or dead cells throughout the life of the animal, and this process is epithelial tissue homeostasis which is maintained through the presence of stem cells^30,31^. The stem cell population were identified in different epithelial tissue including gut, lung and vagina^32–34^. Interestingly, though stem cells reside in the basal layer of the vaginal epithelium of adult mice (and possibly other mammals), only adult females of the hystricomorph rodents periodically form a VCM at the vaginal orifice^34^. Investigating the VCM helps identify where adult stem cells reside within the reproductive tract, and may lead to the discovery of endogenous stem cell populations that could be harvested for autologous therapies, this, in turn, could lead to the development of regenerative “embolics” or membrane materials capable of both sealing defects and promoting healthy tissue remodeling in reproductive tract.

In this study, we compared the characteristics and cellular proliferation patterns of the epithelium surrounding the vaginal orifice in adult female mice and guinea pigs, revealed that progesterone may play a significant role in the formation of the VCM. Furthermore, we identified an epithelial transition zone situated between the epidermis and the vagina in guinea pigs; this region may play a pivotal role in the cyclical formation of the VCM. Moreover, we identified a novel population of stem cells expressing pluripotency markers within the guinea pig. Our findings suggest that, in species belonging to the suborder Hystricomorpha, pluripotent stem cells may reside within the epithelial tissue of the region responsible for VCM formation.

## Results

### Histological structure and hormonal regulation of VCM formation

**Figure 1:**
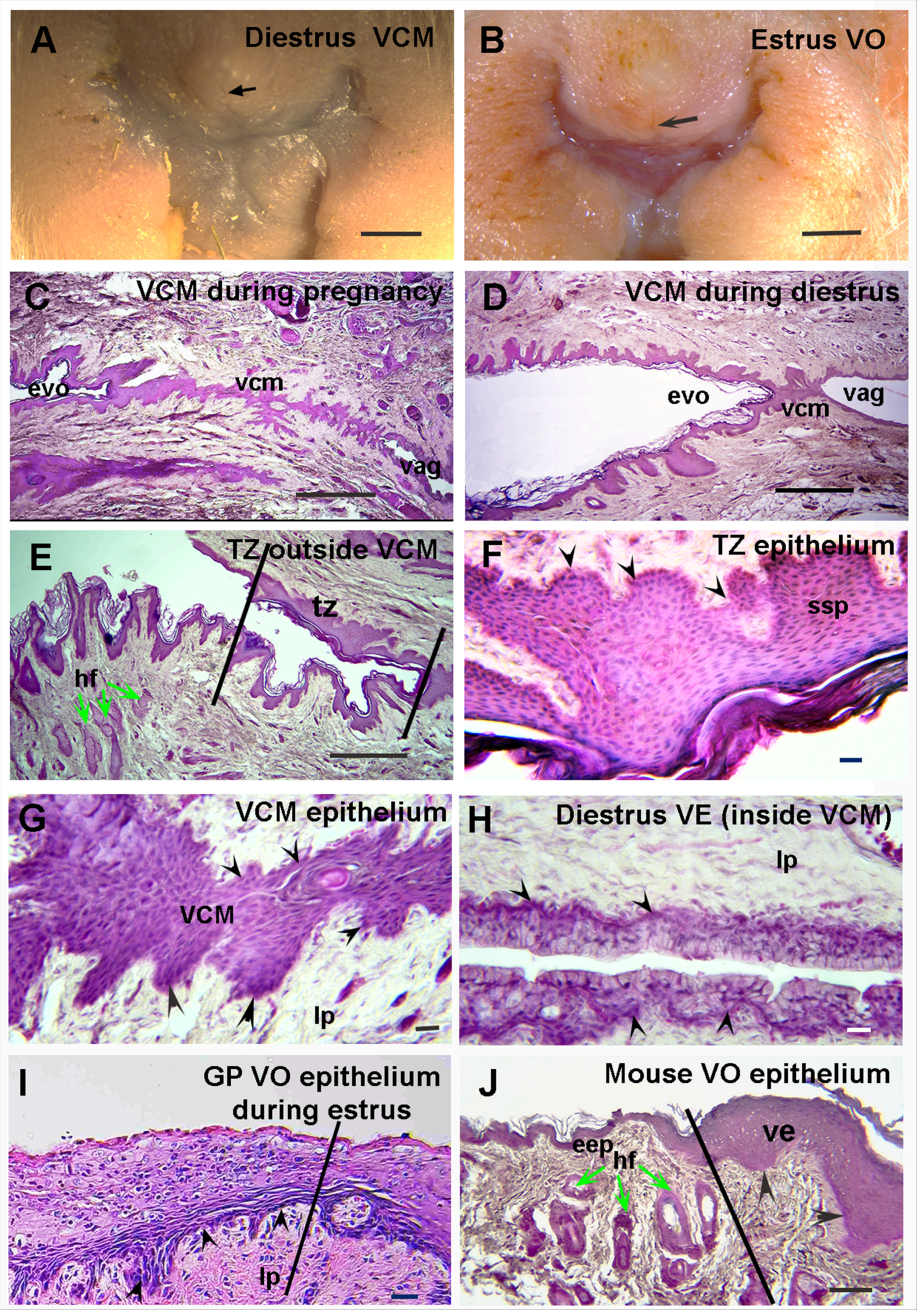
Morphology and structure of VCM and vaginal epithelium (A, B), Ventral views of adult female guinea pig external genital region at diestrus (A) and estrus (B) with the distal of clitoris at the top. Pale VCM completely developed to close the entire vagina in diestrus, black arrows pointat the urethral ostium. (C-J), Sagittal sections of guinea pig (C-I) and mouse (J) vaginawith theexternal vaginal orifice on the left. (C, D) show VCM in pregnant (C) and diestrus (D) guinea pigs. (E, F) show the hairless transition zone epithelium between haired skin and VCM. (G), Structure of VCM. (H), Structure of vaginal epithelium on the inner side of VCM. (I), Structure of vaginal epithelium ofan estrus guinea pig. (J) shows connecting region between the epidermis and vaginal epithelium in a mouse. 2 black lines in (E) indicate the transition zone between keratinized, hairy skin and VCM. Single black line in (I, J) indicates the junction between keratinized, hairy skin and vaginal epithelium. Black arrow heads in (F-J) show basal epithelial layer, green arrows in (E and J) point at hair follicles. eep, epidermal epithelium; evo, epithelium of vaginal orifice; gp, guinea pig; hf, hair follicle; ssp, stratum spinosum; tz, transition zone; vcm, vaginal closure membrane; ve, vaginal epithelium; vo, vaginal orifice. Scale bars: (A, B), 2mm; (C-E), 500μm; (F-J), 10μm

In adult guinea pigs, the vagina opens only during estrus and parturition; at other times, it is occluded by a VCM (Figs. 1A and B). To clearly visualize the VCM, we removed the hair from this region; macroscopically, the VCM appears pale white and is situated at the vaginal orifice—specifically, at the junction between the epidermis and the vaginal epithelium (Figs. 1C and D). The thickness of VCM in adult females varies and the VCM in pregnant animal is about 2mm thick, and it is about 0.5 mm in diestrus (Figs. 1C and D, Table 1). Situated externally to the VCM, an epidermal transition zone lies between the VCM and the haired skin; this transition zone is characterized by a thickening of the germinative layer (basal layer) and the spinous layer, and is devoid of hair (Fig. 1E). The structure and cell types of the VCM resemble those of the transitional zone; indeed, the VCM appears as though two transitional zones have fused together (Figs. 1F and G). In diestrus guinea pigs, the vaginal epithelium on the inner side of the VCM becomes markedly thinned, with only two to three cell layers on top ofthe basal layer separating the underlying connective tissue from the vaginal lumen (Fig. 1H). The vaginal cytological characteristics of guinea pigs during estrus are similar to those of mice and rats, exhibiting a predominance of irregular, anucleated cornified (keratinized) epithelial cells (Fig. 1I and ref^34–36^). The vagina of adult female mice remains continuously open, as is the case in most other animals. And there is no hairless transitional zone between the epidermis and the vaginal epithelium; however, due to the abundance of hair follicles within the epidermis, these two types of epithelia remain clearly distinguishable under a microscope (Fig. 1J).

To determine the influence of ovarian hormones on the formation of the VCM, we performed ovariectomies on guinea pigs at various stages of the estrous cycle. In animals ovariectomized during the diestrus phase, the VCM remained in a closed state (Table 1)—a result consistent with previously published literature^10,37^. If ovariectomy was performed during the estrus phase, the VCM formed by the third postoperative day and the thickness of VCM is similar to diestrus controls (p = 0.1256, Table 1). Conversely, if ovariectomy was performed during the non-estrus phase and estrogen was administered between the 7th and 9th postoperative days, the vaginal orifice was observed to be open upon examination on the 10th day; however, once estrogen injections were discontinued, the VCM reformed within four days. Notably, under these conditions, the VCM formed was thinner than that of the diestrus control group (p = 0.0062, Table 1). If progesterone is administered between days 7 and 9 following ovariectomy in animals in diestrus, the VCM becomes observable upon examination on day 10; by day 15 post-surgery, its thickness reaches 1.85 mm—significantly greater than that of the control group in the diestrus phase (p = 0.0007) and more closely approximating the VCM thickness observed during pregnancy (p = 0.1135). Conversely, if ovariectomized animals are treated concurrently with both estrogen and progesterone, the vagina is observed to be open on day 10 but closed by day 12; although distinct, minute pores remain visible at this stage, the VCM thickness shows no significant difference compared to the control group in the diestrus phase (p = 0.0971, Table 1). The results indicate that progesterone may promote the formation of VCM and maintain its stability, whereas estrogen induces the degradation of VCM.

### The VCM is a partially keratinized and stratified squamous epithelium

**Figure 2:**
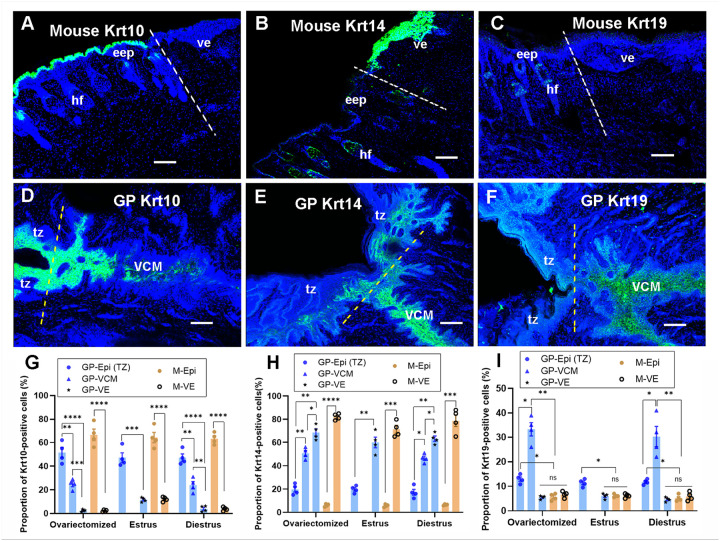
Distribution of different keratins in the epithelium at the external vaginal orifices of mice and guinea pigs (A-F) Immunofluorescence images of Krt10, Krt14, and Krt19 (green) in the epithelium at the external vaginal orifices (sagittal sections) of mice (A–C) and guinea pigs (D–F) during the diestrus phase, oriented with the vaginal orifices facing left (A-F); blue indicates DAPI nuclear staining. (G-I) Quantification of the Krt10- (G), Krt14- (H) and Krt19- (I) positive cells in different tissue of mouse and guinea pigs. *p≤0.05, **p≤0.01, ***p≤0.001, ****p≤0.0001, and ns indicates no significant difference, n=4. The white dashed lines in (A-C) indicate the junction between the epidermis and vaginal epithelium in mice. The yellow dashed lines in (D-F) indicate the junction between the hairless transition zone epithelium and VCM in guinea pigs. epi, epithelium; eep, gp, hf, tz and vcm, ve are the same as in Figure 1. GP and M in (G-I) indicate guinea pig and mouse respectively. Scale bars in (A-F), 100μm.

The main difference between the epidermis and vaginal epithelium lies in the keratinization of the epidermis, while the vaginal epithelium in the anestrus phase mostly does not undergo keratinization andvary with hormones, allowing flexibility and moisture^38,39^. Keratin 10 is commonly used to identify keratinized epithelium^25,40^. In vaginal epithelium of female mice, multiple keratins including keratin 14 is strongly expressed in ovariectomized animals, but keratin 10 and keratin 1 are only expressed in specific stage (such as estrus) and estrogen induction^41,42^. Keratin 19 is usually expressed in non-keratinized basal layer of epithelial cells and a marker for progenitor cells^43,44^. To characterize VCM, we compared the expression of several keratins in the epithelium surrounding the vaginal orifice of diestrus mice and guinea pigs. In mice, our results demonstrate that Krt10 is strongly expressed in the epidermis but is undetectable in the adjacent vaginal epithelium (Fig. 2A); conversely, Krt14 is strongly expressed across multiple layers of vaginal epithelial cells, whereas its expression in the epidermis is confined to hair follicles, with only a few positive cells detected in the epithelial basal layer (Fig. 2B); the expression of Krt19 was observed only in the hair follicles of the epidermal epithelium (Fig. 2C). In guinea pigs, Krt10 exhibits strong expression within the transitional zone and the outer one-third of the VCM, the vast majority of cells are positive; the number of positive cells in the inner portion of the VCM gradually decreases, becoming undetectable at its innermost terminus (Fig. 2D). Krt14 and Krt19 are strongly expressed in epithelial cells throughout both the transitional zone and the entire VCM region (Figs. 2E and F).

To further compare the effects of the estrous cycle on the cellular composition of VCM, we performed a quantitative analysis of the number of Krt10-, Krt14-, and Krt19-positive cells within the vaginal and epidermal epithelia of guinea pigs and mice in the ovariectomized, estrus, and diestrus groups, and the results indicated that, across all three groups, the differences between guinea pigs and mice were highly significant. In the mouse epidermal epithelium, the proportions of Krt10-positive cells exceeded 62% in all the three groups; whereas in the guinea pig transitional zone epithelium (epidermis), these proportions were approximately 50%; and in VCM of guinea pigs in the diestrus and ovariectomized groups, they were approximately 26% (Fig. 2G). In both mice and guinea pigs, the proportions of Krt10-positive cells in the vaginal epithelium in the diestrus and ovariectomized groups were less than 3%, although this proportion rose to over 6% in the estrus group; furthermore, across all three groups, the differences between the two species were not statistically significant (Fig. 2G). In mouse epidermis and vaginal epithelium, the ratios of Krt14 positivity were approximately 4% and 70%, respectively, and no significant differences were observed among the three groups (Fig. 2H). In guinea pigs, the ratios of Krt14-positive cells in the epidermis, VCM, and vaginal epithelium were approximately 20%, 50%, and 63%, respectively in all the three groups; with the exception of the VCM group during the estrus phase (VCM is absent), no significant differences were observed among the remaining groups (Fig. 2H). In mouse epidermis and vaginal epithelium, the proportions of Krt19-positive cells were less than 5% in all the three groups; however, in guinea pigs, they were significantly higher (epidermis: >21%; VCM: ~34%; vaginal epithelium: >11%). Similar to the findings for Krt14, no significant differences were observed in the Krt19-positive cell ratio among the three groups except for the VCM group during the estrus phase (VCM is absent) (Fig. 2I).

### Cell proliferation in the epithelium of transition zone determines the formation of VCM in adult guinea pigs

**Figure 3:**
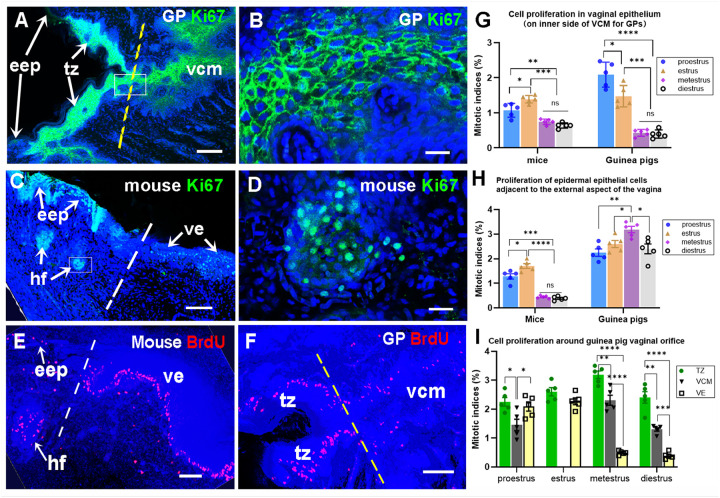
Cell proliferation in the epithelium surrounding the external vaginal orifices of guinea pigs and mice Images in (A–F) display sagittal sections of the external vaginal orifices of guinea pigs (A, B, and F) and mice (C–E) in the diestrus phase, oriented with the vaginal orifices facing left. The green signals in (A–D) indicate the localization of Ki67; the red signals in (E and F) mark BrdU-positive cells. The blue signals represent DAPI nuclear staining. (B) and (D) are magnified images of the respective rectangles in (A) and (C). (G, H) show cell proliferation data (mitotic index—proportion of BrdU-positive cells) for the vaginal epithelium (G) and epidermal epithelium immediately adjacent to the external aspect of the vagina (H) in mice and guinea pigs. (I) show the comparison of cell proliferation in the transition zone (TZ), VCM, and internal vaginal epithelium (VE) of guinea pigs during the proestrus, estrus, metestrus, and diestrus phases. *p≤0.05, **p≤0.01, ***p≤0.001, ****p≤0.0001 and ns indicates no significant difference, n=5. The yellow dashed lines in (A and F) indicate the junction of guinea pig epidermal transition zone and VCM. White dashed lines in (C and E) indicate the junction of mouse epidermis and vaginal epithelium. eep, GP, hf, tz, vcm and ve are the same as in Figure 1. Scale bars: (A, C, E and F), 100μm; (B and D), 25μm.

The reconstruction process of the VCM is relatively rapid; a completely closed VCM can typically be observed two days after the conclusion of the estrus phase. To determine whether increased cellular proliferation occurs during the formation of the VCM, we therefore examined the proliferative activity of the epithelial cells surrounding the vaginal orifice. Ki67 is a widely used marker of cell proliferation; as a nuclear protein, it is expressed during all phases of the cell cycle except for the resting phase (G0)^45^. In guinea pigs during the early diestrus phase, Ki67 exhibited strong expression in the majority of epithelial cells within VCM and transition zone. However, the Ki67 protein was not localized within the nucleus as expected, but rather in the cytoplasm (Figs. 3A and B). In female mice during the diestrus phase, Ki67 was expressed within the nuclei of hair follicle, epidermal, and vaginal epithelial cells (Figs. 3C and D). Subsequently, we employed the 5-bromo-2′-deoxyuridine (BrdU) method^46,47^ to assess cell proliferation during different stages of the guinea pig estrous cycle, and conducted corresponding comparative studies in mice. BrdU-positive cells were detected in the basal layer of the vaginal epithelium/VCM in both mice and guinea pigs (Figs. 3E and F). In mouse epidermis, BrdU-positive cells were also observed in the basal layer and hair follicles (Fig. 3E). Compared with the mice, the highest proportion of BrdU-positive cells in the guinea pig vaginal epithelium (on the inner side of the VCM) was in proestrus (2.24%), followed by estrus phase (1.66%), and the mitotic index in proestrus was significantly higher than that of the same phase in mice (p = 0.0142, Fig. 3G). Subsequently, we calculated the proportion of BrdU-positive cells within the epidermal epithelium immediately adjacent to the outer edge of the VCM (i.e., the transition zone), the results revealed that during the estrus and metestrus phases, the mitotic indices were significantly higher than those observed during proestrus and diestrus (p ≤ 0.0218), with the highest index occurring during metestrus—a period that precisely coincides with the formation phase of the VCM (Fig. 3H). For comparison, we also calculated the mitotic indices of the mouse epidermal epithelium immediately adjacent to the vaginal epithelium, the results indicated that the indices during proestrus and estrus were higher than those during metestrus and diestrus, with the highest level of cell proliferation observed during estrus (Fig. 3H); this result was similar to the pattern of cell division observed in the mouse vaginal epithelium. In guinea pigs, on the other hand, the transition zone exhibited a significantly higher levelof cell proliferation than the VCM and the vaginal epithelium (on the inner side of the VCM) during metestrus and diestrus (p ≤ 0.0026; Fig. 3I) The results suggest the cell proliferation in transition zone may play important roles in VCM formation.

### Pluripotent stem cell markers are expressed in the epithelial cells around the vaginal orifice of guinea pigs, but not of mice.

**Figure 4:**
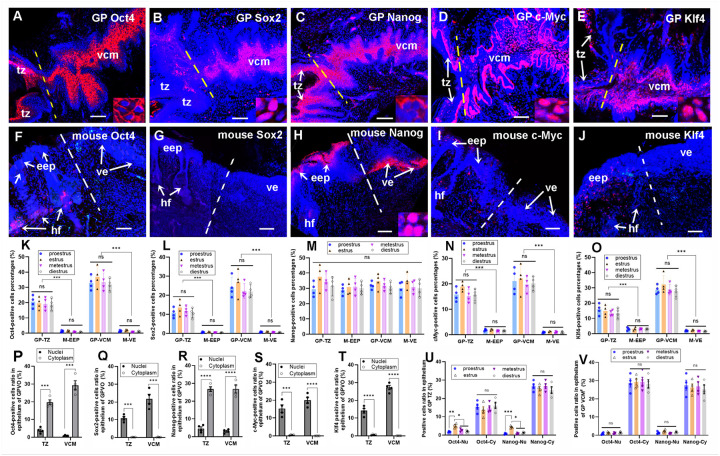
The localization of embryonic stem cell markers Oct4, Sox2, Nanog, c-Myc, and Klf4 in the epithelium at external vaginal orifices of guinea pigs and mice All images in (A-J) represent sagittal sections of the external vaginal orifices of guinea pigs (A-E) and mice (F-J) in the diestrus stage, oriented with the vaginal orifices facing left. Red staining indicates positive cells for Oct4 (A, F), Sox2 (B, G), Nanog (C, H), c-Myc (D, I), and Klf4 (E, J), while blue represents DAPI nuclear staining. The insets in the bottom right corners of (A-E, H) display magnified images of representative cells, illustrating the nuclear or cytoplasmic localization of these marker proteins. (K-O) Quantitative comparison of Oct4 (K), Sox2 (L), Nanog (M), c-Myc (N), and Klf4 (O)-positive cells within the distinct epithelial compartments(transitional zone (TZ), epidermal epithelium (EEP), VCM, and vaginal epithelium (VE)) across the four stages of the estrous cycle in guinea pigs and mice. Notably, none of these five markers exhibited significant differences between the various stages of the estrous cycle. (P-T) Quantitative analysis of the nuclear or cytoplasmic localization of Oct4 (P), Sox2 (Q), Nanog (R), c-Myc (S), and Klf4 (T) within the TZ and VCM of guinea pigs during diestrus phase. Notethat Sox2, c-Myc and Klf4 were predominantly localized within the nucleus, whereas Oct4 and Nanog were primarily localized in the cytoplasm. (U, V) Comparative analysis of the nuclear- or cytoplasmic-localized cell ratios of Oct4 and Nanog within the epithelium of TZ (U) and VCM (V) among the four stages of the estrous cycle. *p≤0.05, **p≤0.01, ***p≤0.001, **** p≤0.0001, ns, no significant difference, n=4. Broken yellow lines indicate the junction between TZ and VCM of GPs. Broken white lines indicate the junction between mouse epidermis and VE. eep, hf, tz, vcm and ve are the same as in Figure 1. Scale bars in (A-J), 100μm. # in (V): Given the absence of VCMs during estrus, we turned to comparing the nuclear and cytoplasmic localization of Oct4 and Nanog in the epithelial cells of the corresponding regions.

Following the conclusion of each estrus in guinea pigs, a VCM forms at the vaginal orifice—a phenomenon not observed in mice. Furthermore, a substantial number of Krt19-positive cells are present within the epithelial tissue surrounding the guinea pig vaginal orifice. Given that Krt19 is widely recognized across various tissues as a marker for progenitor or stem cells^43,48–50^, we hypothesize that a distinct population of stem cells may reside within the vaginal orifice region of guinea pigs. To validate this hypothesis, we performed immunofluorescence staining on the vaginal orifice region tissues of guinea pigs and mice during the diestrus phase to detect the expression of the pluripotency markers Oct4, Sox2, Nanog, c-Myc, and Klf4. In guinea pigs, this analysis revealed that all five proteins exhibited strong expression within both the VCM and the transition zone (Figs. 4A–E). Oct4 was present in the epithelial cells of both the transition zone and the VCM; surprisingly, the vast majority of Oct4 protein was localized within the cytoplasm rather than the nucleus (Fig. 4A). Sox2 was predominantly localized within the nuclei of VCM epithelial cells, with only a small amount distributed in the transitional zone (Fig. 4B). The expression pattern of Nanog was similar to that of Oct4, with positive signal primarily distributed within the cytoplasm of cells in both the VCM and the transitional zone (Fig. 4C). c-Myc and Klf4 were expressed across multiple cell layers, yet positive cells were more abundant within the basal and parabasal layers of both the transition zone and the VCM; furthermore, both of these proteins were predominantly localized within the nucleus (Figs. 4D and E). Compared to guinea pigs, Oct4, Sox2, c-Myc, and Klf4 were not detected in the vaginal epithelium of mice during the diestrus phase; only a small number of positive cells were observed in hair follicles (Oct4 and c-Myc) and the epidermal epithelium (c-Myc and Klf4), and all these signals were localized within the cell nuclei (Figs. 4F, H, I and J). Nanog was strongly expressed in the basal and parabasal layers of the mouse epidermal and vaginal epithelia (Figs. 4H and ref^51^).

To reveal the tissue-specific expression of these transcription factors at different stages of the estrous cycle, we quantified the positive cells in epidermal and adjacent vaginal epithelia. We didn’t find significant differences for all 5 transcription factors among different stages of estrous cycle in guinea pigs and mice (Figs. 4K–O). The positive cell ratios of all factors in the guinea pig epidermal epithelium of the transitional zone and its adjacent region (i.e., outer region) of VCM were significantly higher (p ≤ 0.0004) than those in the corresponding mouse regions except for Nanog, which showed no significant difference between guinea pigs and mice (p ≥ 0.0961, Figs. 4K–O). Interestingly, Oct4 and Nanog were found mainly localized in cytoplasm while Sox2, c-Myc and Klf4 were in nuclei in both epidermal epithelium of the transition zone and the VCM in guinea pigs (Figs. 4A–E), but in mice, Nanog was mainly localized in the nuclei of vaginal epithelium and adjacent epidermal epithelium (Fig. 4H). Quantification of average positive cell ratios of nuclei- and cytoplasm-localized protein revealed that a small portion of Oct4 (3.8%)- and Nanog (4.2%)-nuclei-positive cells were found in the epithelium of guinea pig transition zone, and much higher ratio of Oct4 (14.5%) and Nanog (26.4%) were found in the cytoplasm of the same region (Figs. 4P and R). The cytoplasmic localization of Sox2, c-Myc and Klf4 were very limited (less than 1%, Figs. 4Q, S and T). All the 5 proteins are transcription factors, acting as a core regulatory network that maintains stemness, pluripotency, and self-renewal in stem cells^52^. It was surprising that the majority of Oct4 and Nanog were localized in the cytoplasm and a small portion were also localized in the nuclei in epithelia of guinea pig transition zone and VCM. Then we asked whether the cellular localization of Oct4 and Nanog vary at different stages of the estrous cycle. To answer this question, we compared the nuclear- and cytoplasm-localized cell ratios in epithelia of transition zone and VCM among 4 stages, and revealed that nuclear-localized Oct4 and Nanog were expressed in significantly higher levels at estrus (Oct4, p ≤ 0.0215; Nanog, p ≤ 0.0066) than the other stages in the transition zone, but no significant differences were found in the VCM (p ≥ 0.1522, Figs. 4U and V). Although the majority proteins of Oct4 and Nanog were localized in the cytoplasm, no significant differences in the cytoplasmic expression levels were detected among different stages of the whole estrous cycle in both transition zone and VCM (p ≥ 0.1057, Figs. 4U and V).

## Discussion

The VCM is unique in adult females of hystricomorphic rodents, similar temporary closures of the vaginal orifice occur in some other mammals, but not as a persistent, cyclical breaking and reforming throughout life^4^. Although early researchers hypothesized that the formation of VCM was attributable to post-inflammatory repair processes—involving the participation of plasma cells and fibroblasts—recent studies suggested that the formation of this membrane may result from cellular proliferation within the germinal layer, coupled with mutual adhesion between the cells of the vaginal canal wall^2,12^. Our results indicate that the VCM is composed of both keratinized and non-keratinized epithelial cells. Compared to mice, guinea pigs possess a hair follicle-free transitional zone situated between the epidermis and the vaginal epithelium; cells originating from both this epidermal transitional zone and the vaginal epithelium may contribute to the formation of the VCM. Our findings indicate that VCM is a partially keratinized, stratified squamous epithelium rich in progenitor cells; cell proliferation within the epithelial transition zone is the primary driver of VCM formation. The formation of this membrane is a regenerative process rather than a wound-healing process.

We know that guinea pigs do not menstruate; however, following the conclusion of the estrus phase, the endometrium begins to thicken. The stimulated decidua reaches its peak proliferation between days 10 and 12; subsequently, the thickened portion of the endometrium begins to regress and undergo resorption if they do not conceive^53,54^. Notably, the onset of VCM formation coincides with the commencement of endometrial thickening, and the thickening of both appears to be primarily regulated by progesterone^54^. Whether VCM, like the endometrium, reaches its maximum thickness at a specific time point and subsequently begins to regress requires further investigation.

Remarkably, when detected using the same antibody, Ki-67 was primarily localized in the cytoplasm within the transitional zone of guinea pig epidermis and in VCM; conversely, in mouse epidermis and vaginal epithelium, it was predominantly localized in the nucleus. Ki-67 is a nuclear protein marker essential for measuring cell proliferation in tumors, as it is present during active cell cycle phases (G_1_, S, G_2_ and M), but absent in resting cells (G_0_)^45^. While primarily nuclear, the cell membrane and cytoplasmic pattern of reactivity of Ki67 has been reported in different kinds of invasive tumors^55–57^, moreover, the cytoplasmic expression of Ki67 has also been observed in normal tissue from heart atrial appendages of healthy rats^58^. Clearly, the cytoplasmic expression of Ki67 may be involved in invasion of tumors and normal tissues remodeling. Our results suggest that the VCM formation in guinea pig vaginal orifice is most likely a tissue remodeling process. In addition, the distribution pattern of BrdU-positive cells in VCM is interesting. A relatively large number of positive cells were present near the transition zone, and their abundance gradually decreased along the depth of the sagittal section; this distribution was consistent with that of Krt10-positive cells. These results suggest that cell proliferation in the transition zone may play key roles in the formation of VCM.

It has been demonstrated that the skin of many adult mammals harbors potent stem cell reservoirs within the hair follicle bulge and the dermis; these cells typically express markers such as Oct4, Nanog, and Sox2, and possess the capacity to differentiate into multiple cell lineages, thereby contributing to the promotion of tissue repair and regeneration^59–61^. Our results demonstrated that Oct4, Nanog, Sox2, c-Myc, and Klf4 were predominantly expressed in the epithelial cells of the guinea pig epidermal transitional zone and VCM; furthermore, compared to cells in the corresponding region of mice, the numbers of these positive cells were significantly higher in guinea pigs. This finding suggests that the adult guinea pig vaginal orifice—specifically the region where the VCM forms—contains a greater abundance of multipotent stem cells or progenitor cells than that of the adult mouse. Although Oct4 and Nanog are primarily recognized as nuclear transcription factors that maintain pluripotency, under specific conditions, including embryonic development, somatic cell reprogramming, and tumorigenesis, they can also be detected in the cytoplasm; in such contexts, they may play a role in cell fate, signal transduction, or the regulation of differentiation^62–65^. The cytoplasmic localization of these factors often serves as a key regulatory mechanism for their activity, thereby enabling them to respond to specific developmental signals or cellular stress, rather than merely resulting in their inactivation^66,67^. Clearly, the predominant cytoplasmic localization of Oct4 and Nanog in the guinea pig VCM represents a specific, transient stage of high cell proliferation and active remodeling, rather than a state of pluripotency maintenance. In addition, the nuclear localization of Oct4 was significantly higher in estrus than other phases of the estrous cycle, suggesting that Oct4 is responsive to estrogen, which is consistent to the findings in uterine tissue^68,69^. It is well known that forced expression of the Oct4, Sox2, Klf4, and c-Myc induces somatic cells to dedifferentiate, and overexpression of these four factors results in cellular dedifferentiation, hyperplasia, and teratoma formation in various organs^70,71^. Our findings indicate that the formation of the VCM in guinea pigs constitutes a process of tissue remodeling; furthermore, the overexpression of five pluripotent stem cell factors at the vaginal orifice in adult guinea pigs, but not in mice, may be the primary reason why the phenomenon of periodic VCM formation is observed exclusively in female hystricomorph rodents. Further research is still required to precisely determine how many of these markers are co-expressed within the same cell, and whether the vaginal orifice of adult guinea pigs harbors pluripotent stem cells.

## Materials and methods

### Animals

Adult Hartley guinea pigs were purchased from Elm Hill Labs, and ICR mice were purchased from Envigo RMS Inc (Indianapolis, USA), they were housed in a pathogen-free barrier facility on 12-hour light/dark cycles with access to food and water *ad libitum*, and all experiments were conducted in accordance with the experimental protocols (Guinea pigs: 20 − 014, mice: 23 − 011), which were approved by the Institutional Animal Care and Use Committee of Southern Illinois University Carbondale. To assess the effects of ovarian hormones on VCM formation, we performed bilateral flank-approach ovariectomies on adult female guinea pigs, following the protocol published by Capello V71^72^. Estradiol benzoate and progesterone were purchased from Sigma. Stock solutions of these hormones were prepared using 100% ethanol and subsequently diluted with corn oil to ensure that the ethanol content remained below 0.5% (v/v). From the 7th to the 9th postoperative day, female guinea pigs that had undergone ovariectomy (performed during the diestrus phase) received daily subcutaneous injections of either estradiol benzoate (50 μg/kg), progesterone (200μg/kg), a combination of estradiol benzoate and progesterone, or corn oil containing 0.5% ethanol as a control; daily examinations of the VCM were initiated on the 10th postoperative day. On the 15th postoperative day, VCM samples were collected for histological analysis.

### Estrous cycle stage identification

The estrous cycle stages of guinea pigs were identified by the color and opening and closing state of VCM^3^, the estrous cycle of a female Hartley guinea pig is 16–18 days long. In estrus, the VCM is opened, which usually lasts 1–3 days, then the VCM reforms, the reforming process usually lasts for 1–2 days, this stage is the metestrus, followed by 12–14 days diestrus with a closed VCM. When the membrane starts to change color and become swollen, it reaches the proestrus phase.

Visual and vaginal cytology methods were used to identify the estrous cycle stages of mice^73^. In brief, we first evaluated the estrous cycle stage according to vaginal opening, color, swollen and moist condition and visibility of white cellular debris. Then the cells were collected using a cotton tipped vaginal swab and transferred to a clean glass slide, the wet mount unstained slide was observed using the microscope and the stage of the estrous cycle was determined based on the proportion of leukocytes, cornified epithelial, and nucleated epithelial cells.

### Histology and Immunofluorescence

Adult female guinea pigs and mice of different estrous cycle stages were euthanized, and vaginal orifice tissue were dissected and collected under stereoscope. To detect cell proliferation in guinea pigs and mice, BrdU (50 mg/kg) was injected intraperitoneally and vaginal orifice tissue were collected 4 hours later for immunostaining. After fixation with 4% PFA, paraffin sections were prepared as we described previously^27^ with increased dehydration and clearing time and stained with hematoxylin and eosin. For immunofluorescence staining, vaginal orifice tissues were imbedded using 4% low melting agarose gel (RPI, A20070) and cut into 200 μm sagittal sections on a vibratome. We performed immunohistochemical staining by placing the tissues from both species within the same well of a 24-well plate. This staining procedure employed a standard free-floating immunohistochemistry protocol^74^ modified to suit the specific experimental conditions: given that we utilized relatively thick tissue sections, the incubation times for both the primary and secondary antibodies were extended (48 hours for the primary antibody and 24 hours for the secondary antibody, at 4°C). BrdU immunofluorescence was performed according to standard procedures modified from previously established immunofluorescence protocol^46^ using anti-BrdU (G3G4, DSHB, Cat# AB 2314035, RRID: AB_2618097). Sample size, n = 4. The immunofluorescence of Ki67, Krt10, Krt14 and Krt19 in guinea pig and mouse vaginal orifice tissue sections was performed using anti-Ki67/MKl67 (NOVUS, Cat# NB500–170, RRID:AB_10001977), anti-Krt10 (Abcolonal, Cat# A4609, RRID: AB_3741489), anti-Krt14 (BioLegend, Cat# 905304, RRID:AB_2616896) and anti-Krt19 (Proteintech, Cat#10712–1-AP, RRID:AB_2133325).

Immunofluorescence of the stem cell markers Oct4, Sox2, Nanog, c-Myc and Klf4 in guinea pig and mouse vaginal orifice tissue sections was performed with Tyramide SuperBoost Kits (Invitrogen, Cat# B40922&B40926) following the user guide using anti-OCT4/POU5F1 (proteintech, Cat# 11263–1-A*P*, RRID:AB_2167545), anti-SOX2 (proteintech, Cat# 11064–1-AP, RRID:AB_2195801), anti-NANOG (proteintech, Cat# 14295–1-AP, RRID:AB_1607719), anti-c-MYC (proteintech, Cat# 10828–1-AP, RRID:AB_2148585) and anti-KLF4 (proteintech, Cat# 11880–1-AP, RRID:AB_10640807). Sample size, n = 5.

### Stereology and quantification

Several vibratome sections from different animals (n = 5) were performed for immunostaining of BrdU, keratins and stem cell markers, and stereology was used to determine the mitotic indices and protein-positive cell ratios in the epidermal and vaginal epithelium within the vaginal orifice regions of mice and guinea pigs. The number of DAPI-labeled (total), BrdU-, keratin- or stem cell marker-labeled cells were estimated stereologically using the optical fractionator method under a confocal microscope by counting target cells on every 5th 3-μm transverse optical section (about 10 (mice) and 20 (guinea pigs) sections from the center of sagittal plane were used) following published methods for counting cells through irregularly shaped structures^46,75,76^. Briefly, cells of interest were counted on image stacks acquired using a 20x objective, with Image-J software. Cells that fell within the 30×30-μm counting frames were used in the analyses. The method for calculating cell proportions is as follows: within at least three independent counting frames located in various regions of the tissue section (specifically, the vaginal epithelium), both the total number of cells (labeled with DAPI) and the cells labeled with antibodies for BrdU, keratin, or stem cell markers are enumerated. For the transitional zone in guinea pigs, however, labeled epithelial cells are counted across the entire area of the tissue section to avoid errors arising from localized sampling.

### Statistical Analysis

Comparisons between two groups were performed using a two-tailed nonparametric t-test; comparisons among multiple groups were performed using one-way analysis of variance (ANOVA) with a Turkey-Kramer post hoc test. Quantitative data in all graphs and tables are presented as the mean ± standard error (SE). All analyses were performed using GraphPad Prism 10 software. A p-value ≤ 0.05 was considered statistically significant.

## Figures and Tables

**Table 1:** The effect of ovarian hormones on VCM formation

Adult females	Proestrus 0.35mm (+)*		metestrus closing (+/−)	Diestrus (c) 0.52mm (+)		Estrus No VCM (−)	
Pregnant	27 days pregnant 1.82mm (++)***		30 days pregnant 1.88mm (++)***	45 days pregnant 1.92mm (++)***		Day of birth No VCM (−)	
females							
OE females	24h after OE	48h after OE	5 days after OE	7–9 days after OE	10 days after OE	12 days after OE	15days after OE
OE at diestrus (c)	+	+	0.56mm	+	+	+	**0.61mm**
OE at estrus		+/−	0.36mm*	+	+	+	0.39mm*
OE at diestrus	+	+	0.62mm	E2 once daily		+/−	0.27mm**#
OE at diestrus	+	+	0.55mm	P4 once daily	++	++	1.45mm***
OE at diestrus	+	+	0.65mm	E2 + P4 once daily		+/−	0.34mm*#

VCM thickness measurements, accompanied by numerical labels, were derived from sagittal sections (n = 3); those without numerical labels were obtained through observation of live animals illuminated by flashlight (n = 3). For female guinea pigs without an ovariectomy, the VCM thickness during diestrus (0.52mm) was used as the control and compared with the thickness at other stages of the estrous cycle, as well as during pregnancy. For ovariectomized females, the control group consisted of animals that underwent ovariectomy during diestrus and received the vehicle (0.61mm).

A single “+” indicates a thickness of less than 1 mm, while a double “++” indicates a thickness greater than 1 mm; “−” indicates the absence of a vaginal closure membrane (VCM) and an open vagina; “+/−” indicates an incompletely formed VCM with the vagina in a semi-open state. The hash symbol (#) indicates that the VCM appeared closed in the live animal, yet minute perforations were detected.

* p ≤ 0.05,

** p ≤ 0.01

*** p ≤ 0.001. E2, estradiol; OE, ovariectomy; P4, progesterone.

## Data Availability

All data are available upon reasonable request to the corresponding author.
